# Maternal prenatal blood mercury is not adversely associated with offspring IQ at 8 years provided the mother eats fish: A British prebirth cohort study

**DOI:** 10.1016/j.ijheh.2017.07.004

**Published:** 2017-10

**Authors:** Jean Golding, Joseph R. Hibbeln, Steven M. Gregory, Yasmin Iles-Caven, Alan Emond, Caroline M. Taylor

**Affiliations:** a Centre for Child and Adolescent Health, School of Social & Community Medicine, University of Bristol, Oakfield House, Oakfield Grove, Bristol BS8 2BN, UK; b Section on Nutritional Neurosciences, National Institute on Alcohol Abuse and Alcoholism, National Institutes of Health,5625 Fishers Lane, Rm 3N-07, MSC 9410 Bethesda, MD 20892, USA

**Keywords:** ALSPAC, Mercury, Fetal exposure, Childhood IQ, Prenatal fish consumption

## Abstract

**Background:**

Conflicting evidence concerning possible harm from mercury (Hg) in regard to offspring cognition if the woman eats fish has prompted this study to examine evidence from a British pre-birth cohort to investigate the relationship between the two.

**Methods:**

Pregnant women (median prenatal blood mercury 1.86 μg/L) resident in the study area with delivery between April 1991 and December 1992 were followed up and verbal, performance and total intelligence quotient (IQ) of 2062 offspring were measured at age 8. Analysis treated IQ as (a) continuous and (b) the lowest 25% of the distribution. Multiple and logistic regression analyses took account of social and demographic variables. Stratification considered children of fish eaters separately.

**Results:**

Before adjustment, mean full-scale IQ increased with increasing Hg (change with 1SD of Hg = +2.02; 95%CI +1.40,+2.64 IQ points; P < 0.0001); after adjustment effect size was reduced although still positive (+0.61;95%CI -0.06,+1.29 IQ points; P = 0.073). The adjusted positive relationship was stronger when fish-eating mothers were considered separately (+0.84:95%CI +0.13,+1.56 IQ points; P = 0.021) in comparison with the outcomes for non-fish eaters, where the adjusted relationship was negative (-2.22;95%CI -5.00,+0.56 IQ points; P = 0.117). The binary outcome showed a similar pattern with the adjusted OR for non-fish-eaters 1.79 (95%CI 1.10,2.93; P = 0.019) per SD of Hg, significantly different from that for fish consumers (0.94;95%CI:0.82,1.08)(P_interaction_<0.05). There were no differences between the sexes in the associations, nor did the level of the mother’s blood selenium change the effect sizes.

**Conclusion:**

The relationship between intrauterine exposure to mercury and offspring IQ appears to be benign provided the mother consumes fish.

## 1 Introduction

Cognition, measured as an intellectual quotient (IQ), is one of the fundamental attributes associated with educational and occupational achievements. Economists estimate that the earning power of an individual increases by 2% for each increase in a single IQ point with, at the population level, consequent increases in the annual income of nations − that of the USA is measured in hundreds of billions of dollars (Grosse et al., 2002). Consequently it is in the interest of all nations to maximize the mean IQ of their population by fostering the environmental components that improve IQ while reducing those that diminish it.

Epidemiologists and risk assessors have struggled to interpret the scientific evidence pertaining to mercury in seafood as it relates to cognition. Although there is strong evidence from major accidents involving mercury contamination demonstrating serious offspring brain damage resulting from prenatal exposure to very high levels of mercury (Harada, 1968), a number of studies have shown positive benefits to the neurocognition of the offspring if the mother consumes fish prenatally in spite of the fact that fish contains mercury (Hibbeln et al., 2007, Oken et al., 2005, Mendez et al., 2009, Starling et al., 2015). Advice to pregnant women tends to be confusing, and their reaction appears to be dominated more by the fear of exposure to mercury than the benefits of fish.

The analyses in this paper are designed to provide further information to help address the scientific argument. We use the Avon Longitudinal Study of Parents and Children (ALSPAC) (Boyd et al., 2013), which is the largest study to date to have relevant information available. The aims of our analyses of these data are to determine whether: (a) prenatal blood mercury levels were associated with either lower mean offspring IQ or increased risk of low level IQ; (b) maternal seafood consumption or blood selenium levels offset any negative effects of maternal mercury in regard to offspring IQ, and (c) results are sex-specific.

## 2 Methods

### 2.1 The participants

The ALSPAC study aimed to enroll all pregnant women residing in Avon (a geographically defined area in South West England) with an expected delivery date between April 1991 and December 1992. The study enrolled 14,541 pregnant women, estimated as about 80% of those eligible. Its stated aims were to evaluate genetic and environmental influences on health and development (Boyd et al., 2013).

Information was collected prenatally using self-completion questionnaires sent to the mothers and their partners in their own homes and blood was collected at their first antenatal visit. The study website contains details of all the data collected subsequently that are available through a fully searchable data dictionary: http://www.bris.ac.uk/alspac/researchers/data-access/data-dictionary/

### 2.2 The exposures

#### 2.2.1 Trace metal exposures

Blood samples collected in acid-washed containers specifically for determination of trace metals were obtained from 4484 women by midwives as early as possible in pregnancy. The sociodemographic characteristics of the women who donated samples were mostly comparable to those of the rest of the ALSPAC study population although they were slightly more likely to be older and better educated (Taylor et al., 2013). Gestational age at sample collection had a median value of 11 weeks and mode of 10 weeks. The interquartile range (IQR) was 9–13 weeks, and 93% of the samples were collected at <18 weeks gestation. Samples were stored as whole blood in the original collection tubes for 18–19 years. Analyses were carried out in the laboratory of Dr. Robert Jones at the Centers for Disease Control and Prevention (CDC) for whole blood mercury and selenium (CDC method 3009.1; unpublished information). Further details are given elsewhere (Golding et al., 2013). The distributions were approximately normal for selenium and slightly skewed for mercury. There were 4134 women with recorded mercury levels which ranged from below the limit of detection (LOD) (0.24 μg/L) to 12.76 μg/L, with a median of 1.86 μg/L. For the three individuals below the LOD, a value of 0.7 times the LOD value was ascribed to the sample. Valid levels of selenium were available for 4287 pregnancies. The range of selenium levels was from 17.0 to 324.1 with median 108 μg/L; no samples were below the LOD.

#### 2.2.2 Maternal diet

A questionnaire sent to the mother at 32 weeks gestation included a food frequency questionnaire comprising 103 food and drink items including two items related to fish intake: white fish and oily fish (Rogers and Emmett, 1998). The participants were given guidelines to classify the types of fish from those that were most prevalent in the UK. Thus oily fish was described as including ‘salmon, mackerel, sardines, trout, herring, pilchards, tuna, etc.’; white fish as including ‘cod, haddock, plaice, fish fingers, etc.’ The woman was asked approximately how frequently she was currently eating each type of fish, with options: ‘not at all; about once in two weeks; 1–3 times a week; 4–7 times a week; more than once a day’. Non-fish-eaters were defined as recording ‘not at all’ for both white and oily fish.

### 2.3 The outcomes

At 8 years of age, the Wechsler Intelligence Scale for Children WISC-III ^UK^ was used to assess cognitive function (Wechsler et al., 1992). A short form of the measure was employed in ALSPAC, administered in a specially designed clinic by trained psychologists as described elsewhere (Bath et al., 2013). The mean (SD) of the full-scale, verbal and performance subtests calculated in this way were: 104.0 (16.5); 107.0 (16.8); 99.5 (17.1) IQ points, respectively, and each was approximately normally distributed.

### 2.4 Possible confounders

We allowed for the following social factors: a continuous family adversity score which is derived from 38 factors present in pregnancy including maternal depression and anxiety; housing tenure (public housing v. rest); household crowding (no. of persons in household divided by the number of rooms available); stressful life events in first half of pregnancy (sum of 44 possible events − treated as continuous scale); smoking at mid-pregnancy (yes v. no); alcohol consumption mid-pregnancy (yes v. no); maternal age at birth; parity (no. of previous deliveries); maternal education (measured on a 5-point scale). Since the IQ measure varied with age and sex of the child, these factors were also taken into account. We did not allow for birthweight or gestation as we consider these to be likely to be on a common pathway to IQ. Since there is considerable evidence that breast feeding has a beneficial effect on the child’s IQ, and there is also evidence that the type of mother who chooses a good diet with the inclusion of fish is one who also chooses to breast feed, this indicates that breast feeding should be taken into account, regardless of whether a mediator or a confounder. If this is inappropriate then the real benefits of mercury levels will have been reduced in size, but it is unlikely to hide adverse effects.

### 2.5 Statistical analyses

Multiple regression was used for IQ measured as continuous, and logistic regression for the lowest quartile of the score. Results are given for Model A (allowing for age at testing and sex); and Model B (additionally allowing for the confounders). Model C additionally also allowed for selenium as a sensitivity analysis to determine whether a moderator. The analyses were repeated according to whether the mother had eaten fish or not prenatally and tested for interaction; a similar exercise tested whether there was a sex difference. Since we do not consider that the data are missing at random, we have not included analyses concerning missingness. The analyses were carried out using STATA (version 14.1).

## 3 Results

### 3.1 Unadjusted analyses

We have shown elsewhere that there were no differences between the women for whom a trace metal result was obtained compared with the rest of the population in relation to their seafood intake or dental treatment (Golding et al., 2013, Golding et al., 2016). In Table 1 we also show that there was no bias in regard to a variety of social conditions and lifestyles, except that older and/or more educated women were more likely to have had blood taken for trace metal analyses. Elsewhere we have shown that the mothers who ate fish had mean mercury levels which rose with the frequency of intake (Golding et al., 2013).

**Table 1:** Characteristics of sample of mothers with data on blood mercury levels compared with the rest of the ALSPAC cohort.

Variable	Mothers with blood Hg n (%)	Rest of ALSPAC cohort n (%)	*p* value (chi square test)
n	4285 (29.5)	10257 (70.5)	
Age (years)			
<20	240 (6.1)	628 (6.5)	0.002
≥20–24	719 (18.2)	1935 (20.2)	
≥25–29	1537 (38.9)	3799 (39.6)	
≥30–34	1105 (28.0)	2389 (24.9)	
≥35	346 (8.8)	847 (8.8)	

Maternal education			
None/CSE	709 (19.2)	1785 (20.6)	<0.001
Vocational	345 (9.4)	870 (10.1)	
O level	1226 (33.3)	3047 (35.2)	
A level	841 (22.8)	1930 (22.3)	
Degree	566 (15.4)	1021 (11.8)	
Spent whole life in Avon			0.132 (NS)
No	1859 (47.6)	4379 (46.2)	
Yes	2044 (52.4)	5099 (53.8)	

Maternal social class			
I	200 (6.6)	391 (5.6)	0.383 (NS)
II	960 (31.7)	2182 (31.3)	
III (non-manual)	1276 (42.2)	2998 (43.0)	
III (manual)	228 (7.5)	555 (8.0)	
IV	360 (9.7)	843 (9.9)	
V	67 (2.2)	153 (2.2)	
Ethnicity			0.131 (NS)
White	3585 (97.6)	8342 (97.3)	
Black (African, Caribbean, other)	42 (1.1)	87 (1.0)	
Indian, Pakistani, Bangladeshi	23 (0.6)	60 (0.7)	
Other	22 (0.6)	87 (1.0)	

Housing			
Mortgaged/owned	2827 (72.7)	6930 (73.4)	0.426 (NS)
Rented/other	1062 (27.3)	2516 (26.6)	
Maternal smoking in pregnancy			
Yes	985 (25.3)	2276 (24.6)	0.397 (NS)
No	2905 (74.7)	6968 (75.4)	

Paternal smoking in pregnancy			
Yes	1411 (37.9)	3321 (37.5)	0.641 (NS)
No	2310 (62.1)	5540 (62.5)	

Parity			
0	1684 (44.5)	4085 (45.4)	0.276 (NS)
1	1303 (34.4)	3132 (34.8)	
2	554 (14.6)	1261 (14.0)	
3	176 (4.7)	350 (3.9)	
>3	67 (1.8)	162 (1.9)	

The unadjusted mean IQ levels stratified by maternal prenatal mercury levels are shown in Table 2. For verbal, performance and total IQ, the mean values increased with increasing mercury level (P<0.001). The mean IQs of offspring whose mothers’ blood mercury levels were in the highest 10% of the distribution (>3.39 μg/L) were higher than those in any other category.

**Table 2:** Mean (SE) IQ levels according to maternal prenatal mercury levels divided at the 20th, 40th, 60th, 80th, and 90th centiles.

			Mean (SE)	
Mercury μg/L	N	Verbal IQ	Performance IQ	Total IQ
All children				
<1.28	321	104.4 (0.874)	96.4 (0.898)	100.9 (0.857)
1.28−1.68	394	105.2 (0.867)	98.5 (0.878)	102.4 (0.851)
1.69−2.10	424	108.3 (0.845)	100.5 (0.800)	105.3 (0.798)
2.11−2.74	442	108.3 (0.777)	101.7 (0.777)	106.0 (0.752)
2.75−3.39	239	110.7 (1.086)	99.1 (1.097)	106.1 (1.051)
> 3.39	244	111.7 (1.038)	103.5 (1.138)	108.9 (1.060)
P*		<0.0001	<0.0001	<0.0001

Children whose mothers ate fish				
<1.28	198	103.8 (1.116)	94.9 (1.142)	99.8 (1.095)
1.28−1.68	310	105.9 (0.958)	98.4 (0.966)	102.7 (0.934)
1.69−2.10	376	109.0 (0.889)	100.8 (0.851)	105.9 (0.841)
2.11−2.74	398	108.8 (0.814)	101.9 (0.808)	106.4 (0.780)
2.75−3.39	219	110.9 (1.135)	99.3 (1.150)	106.4 (1.102)
> 3.39	230	111.7 (1.068)	104.1 (1.176)	109.3 (1.100)
P*		<0.0001	<0.0001	<0.0001

Children whose mothers ate no fish				
<1.28	102	106.0 (1.518)	99.6 (1.534)	103.3 (1.456)
1.28−1.68	57	103.7 (2.384)	99.7 (2.658)	102.4 (2.534)
1.69−2.10	31	105.2 (3.297)	99.6 (2.962)	103.0 (3.109)
2.11−2.74	29	105.8 (3.154)	101.6 (3.224)	104.1 (3.193)
2.75−3.39	8	108.3 (5.028)	88.6 (4.196)	98.5 (4.149)
>3.39	5	111.2 (6.272)	95.2 (2.973)	104.6 (5.006)
P*		0.898	0.538	0.971

*P for trend.

Stratification by maternal fish consumption, however, revealed contrasting trends: the offspring of women who did not eat fish had mean IQ levels that stayed roughly the same the higher the mercury level, whereas for the offspring of fish-eaters there was an increasing level of IQ with increasing blood mercury (Table 2).

### 3.2 Adjusted analyses

Table 3 shows the regression coefficient of the mother’s prenatal mercury with the child’s IQ after minimal adjustment (age and sex), followed by additional adjustment for social and demographic factors (Model B). A further model allowed for selenium but the results were essentially the same (data not shown). For all children, regardless of whether or not their mother ate fish in pregnancy, the child’s full-scale IQ shows a positive association with maternal mercury with an increase of +2.02 (95% CI +1.40,+2.64) IQ points per standard deviation (SD) of blood mercury after allowing for age and sex (P < 0.0001). This association reduced dramatically on adjustment for social factors, although the relationship remained positive (0.61[95%CI -0.06, +1.29]; P = 0.073). Similar positive associations are shown for performance and verbal IQ. Adjustment for selenium level had little effect on the regression coefficients.

**Table 3:** Relationship (change in IQ for each SD of mercury) between prenatal mercury exposure and the child’s IQ score at age 8 after adjustments (see footnote below). Model A controls for child’s sex and age at testing; Model B in addition controls for family adversity index, housing tenure, household crowding, stressful life events in first half of pregnancy, smoking at mid-pregnancy, alcohol intake at mid-pregnancy, maternal age, parity and whether the infant was breast fed.

	All children		Mother ate Fish		Mother ate no fish	
Measurement	N	β [95% CI]	N	β [95% CI]	N	β [95% CI]
Verbal IQ						
Model A	2066	+2.03 [+1.39,+2.67]	1732	+1.92 [+1.21,+2.62]	233	+1.19 [−1.76,+4.13]
Model B	1783	+0.52 [−0.17,+1.21]	1562	+0.62 [−0.11,+1.36]	201	−1.31 [-4.10,+1.49]

Performance IQ						
Model A	2065	+1.46 [+0.82,+2.11]	1732	+1.60 [+0.89,+2.32]	232	−0.67 [-3.66,+2.33]
Model B	1783	+0.53 [−0.19,+1.25]	1563	+0.86 [+0.09,+1.62]	200	−2.91 [-5.94,+0.12]^a^

Full-scale IQ						
Model A	2062	+2.02 [+1.40,+2.64]	1729	+2.03 [+1.35,+2.72]	232	+0.40 [−2.50,+3.29]
Model B	1780	+0.61 [−0.06,+1.29]	1560	+0.84 [+0.13,+1.56]	200	−2.22 [-5.00,+0.56]^a^

a Interaction between fish and non-fish eaters P < 0.05.

For children of women who had eaten fish, the results were similar (second column of Table 3) but with slightly larger effect sizes; the results for full-scale IQ remained statistically significant even after adjustment (+0.84[95% CI +0.13, +1.56]; P = 0.021). However, for the children born to the women who had not eaten fish, the relationships were negative after adjustment − i.e. the higher the maternal level of mercury, the lower the mean IQ. The differences in effect sizes between the fish eaters and the non-fish eaters were statistically significant for full-scale IQ (P = 0.043) and performance IQ (P = 0.021) although not for verbal IQ (P = 0.238).

Logistic regression analyses concerning the risk of a low offspring IQ (lowest quartile) indicate that, for all children analyzed together, there was no association after adjustment; a similar lack of association was found if the mothers ate fish (adjusted OR 0.94 [95%CI 0.82,1.08] P = 0.374); however, with increasing mercury, for maternal non-fish-eaters the offspring was more likely to have a low IQ (adjusted OR 1.79 [95% CI 1.10, 2.93] P = 0.019), significantly different from the finding for the children of fish-eaters (Table 4).

**Table 4:** Relationship between prenatal mercury exposure and the child’s having an IQ score at age 8 in the lowest quartile after adjustments (see footnote below). Model A controls for child’s sex and age at testing; Model B in addition controls for family adversity index, housing tenure, household crowding, stressful life events in first half of pregnancy, smoking at mid-pregnancy, alcohol intake at mid-pregnancy, maternal age, parity and whether the infant was breast fed.

	All children		Mother ate Fish		Mother ate no fish	
Measurement	N	OR [95% CI]	N	OR [95% CI]	N	OR [95% CI]
Verbal IQ						
Model A	2062	0.74 [0.67, 0.83]	1729	0.72 [0.64, 0.82]	232	1.12 [0.76, 1.65]^a^
Model B	1758	0.94 [0.83, 1.07]	1555	0.89 [0.78, 1.02]	198	1.64 [0.95, 2.83]^a^

Performance IQ						
Model A	2062	0.87 [0.79, 0.96]	1729	0.85 [0.76, 0.95]	232	1.14 [0.77, 1.68]
Model B	1758	1.01 [0.90, 1.13]	1555	0.99 [0.88 1.11]	198	1.40 [0.90, 2.18]

Full-scale IQ						
Model A	2062	0.77 [0.70, 0.86]	1729	0.75 [0.67, 0.85]	232	1.25 [0.85, 1.84]^a^
Model B	1758	1.00 [0.89, 1.13]	1555	0.94 [0.82, 1.08]	198	1.79 [1.10, 2.93]^a^

a Interaction between fish and non-fish eaters P < 0.05.

Further analyses to determine whether there was a difference in effect size between children of women who ate fish only once in a two weeks and those who ate fish more often showed no differences (data not shown). In addition we determined whether the protective effect was more apparent for oily as opposed to white fish, but no differences were found (data not shown).

Further analysis to assess whether there were differences in results of boys compared with girls (Table 5A, Table 5B) demonstrated that the female offspring were at increased risk of low IQ if their mothers had not consumed fish in pregnancy (OR = 2.32[95%CI 1.10, 4.88] P 0.027), a result significantly different from the risk to offspring of the fish-eaters (P < 0.05); however the results for girls were not significantly different from those for boys (OR 1.63; 95%CI 0.80,3.30).

**Table 5A:** Relationship between prenatal mercury exposure and the boy’s IQ score at age 8 in the lowest quartile after adjustment for A (age at assessment) and B (A plus maternal age, parity and education), comparing offspring of women who ate fish with those who did not.

	VERBAL IQ		PERFORMANCE IQ		FULL-SCALE IQ	
	N	OR [95% CI]	N	OR [95% CI]	N	OR [95% CI]
All boys						
Model A	1026	0.73 [0.63, 0.86]	1026	0.87 [0.76, 1.00]	1026	0.81 [0.70, 0.94]
Model B	878	0.93 [0.77, 1.11]	878	0.97 [0.82, 1.13]	878	1.01 [0.85, 1.19]

Boys whose mothers ate fish						
Model A	867	0.75 [0.63, 0.90]	867	0.85 [0.73, 0.99]	867	0.82 [0.70, 0.97]
Model B	773	0.90 [0.74, 1.10]	773	0.96 [0.81, 1.14]	773	0.98 [0.82, 1.17]

Boys whose mothers ate no fish						
Model A	120	1.06 [0.62, 1.79]	120	0.96 [0.56, 1.64]	120	1.04 [0.61, 1.79]
Model B	103	1.87 [0.86, 4.06]	103	1.22 [0.64, 2.31]	103	1.63 [0.80, 3.30]

**Table 5B:** Relationship between prenatal mercury exposure and the girl’s IQ score at age 8 in the lowest quartile after adjustment for A (age at assessment) and B (A plus maternal age, parity and education), comparing offspring of women who ate fish with those who did not.

	VERBAL IQ		PERFORMANCE IQ		FULL-SCALE IQ	
	N	OR [95% CI]	N	OR [95% CI]	N	OR [95% CI]
All girls						
Model A	1036	0.75 [0.65, 0.88]	1036	0.87 [0.76, 1.00]	1036	0.74 [0.63, 0.86]
Model B	881	0.97 [0.80, 1.16]	880	1.05 [0.90, 1.23]	880	1.01 [0.84, 1.21]

Girls whose mothers ate fish						
Model A	862	0.70 [0.58, 0.84]	862	0.85 [0.73, 0.99]	867	0.68 [0.57, 0.82]
Model B	782	0.90 [0.73, 1.09]	782	1.02 [0.86, 1.21]	782	0.92 [0.75, 1.12]

Girls whose mothers ate no fish						
Model A	112	1.16 [0.65, 2.09]	112	1.35 [0.77, 2.37]	112	1.50 [0.86, 2.61]^a^
Model B	96	1.71 [0.67, 4.38]	95	1.71 [0.89, 3.30]	95	2.32 [1.10, 4.88]^a^

a Interaction between fish and non-fish eaters P < 0.05.

## 4 Discussion

### 4.1 The findings

This study was concerned with more mother-child dyads than in any other study looking at the relationship between prenatal mercury exposure and child IQ. We have shown strong positive associations between maternal prenatal blood mercury and offspring IQ in unadjusted regression analyses. Effect sizes were attenuated after adjustment for social and demographic factors but taken as a whole there was no adverse relationship between prenatal mercury levels and mean IQ. When the offspring of the women who ate fish during pregnancy were considered separately from those of women who did not eat fish contrasting effects were found: there was a negative relationship between the prenatal mercury level and offspring IQ in the non-fish eaters but a positive association among the offspring of the fish-eaters. A similar finding was shown for the risk of being in the lowest quartile of the IQ distribution. Both interactions of the effects between fish and non-fish eaters were statistically significant.

The initial aims of this study were to determine whether there was a negative association overall between prenatal blood mercury levels and offspring IQ. Before adjustment there were strong positive (rather than negative) associations between maternal prenatal blood mercury and offspring mean IQ, but the effect sizes were diminished after adjustment for social and demographic factors. However, there were no adverse relationships between prenatal mercury levels and mean offspring IQ, even considering a P value of 0.10. The second aim was to assess whether there were differing results between women who did and did not eat fish. Even though the numbers of women in this population who never ate fish were relatively low, we found significant differences in the results between the two groups (Table 3, Table 4) such that the offspring of fish eating women had higher mean IQs and lower risk of suboptimal IQ level with increasing blood mercury levels, but the offspring of women who did not eat fish had worse outcomes related to prenatal mercury exposure. Thus for full-scale IQ the difference between the two groups after adjustment for Model B is about 3 IQ points per SD of mercury. This is reflected in the results for the risk of suboptimal IQ where the risk for infants of non-fish eating mothers being in the lowest quartile of IQ is 1.79 [95% CI 1.10, 2.93].

A supplementary aim was to assess whether the mother’s prenatal blood level of selenium had any protective effect on relationships between mercury and child IQ since this has been suggested as the mechanism for the beneficial effect of prenatal fish consumption (fish provides a major source of selenium in the diet) (Berry and Ralston, 2008). This was tested by allowing for selenium in the analyses, but this resulted in only marginal differences in the relationships between mercury and offspring IQ (Table 3, Table 4).

Our final aim was to assess whether results differed between boys and girls. Although there were no statistical differences between the results for the two sexes we were able to show that the increased risk of sub-optimal IQ was particularly strong for the girls of non-fish eating mothers.

### 4.2 Strengths and limitations

The strength of these results lies in the design of the study, covering a geographically defined area with long-term follow-up. The study involves larger numbers than other studies with both prenatal mercury measures and offspring IQ. It also benefits from including a population of pregnant women who ate no fish during pregnancy and having a measure of mercury in the first half of pregnancy, a contrast with most studies where the measure is umbilical cord or maternal hair (e.g. Seychelles Study (Davidson et al., 2006); Faroes cohort (Debes et al., 2006); INMA cohort in Spain (Llop et al., 2016) Project Viva in Boston (Oken et al., 2005)).

It should be noted that the ALSPAC pregnancies occurred prior to official advice on the potential adverse effects of eating some types of fish in pregnancy.

There are a number of possible limitations. First, although the analyses allowed for many possible confounders, there may well be other pertinent factors that were not considered here. Nevertheless the same set of social factors were taken into account among the non-fish consumers as among the fish consumers, and thus it is difficult to explain the difference in results between the two groups. Second, the measure of mercury was obtained from whole blood that had been stored for 18+ years; we think this is unlikely to be a problem since the analysis was undertaken by an experienced laboratory and data on relationships of mercury levels with diet and dental amalgam are in line with expectations (Golding et al., 2013, Golding et al., 2016). Third, the IQ measure used an alternate item strategy rather than the standard WISC format. As a separate validation exercise we have used the full item WISC administered at age 8 elsewhere (Goodfellow et al., 2013), and selected the alternate items in that scale; comparing the resulting IQ with the full IQ measure gives excellent correlation coefficients of 0.965, 0.954 and 0.943 for the three scales, respectively. Fourth, the information on the mother’s diet was obtained in the third trimester, several months after the blood sample was taken. However, Northstone and Emmett have shown that over time the dietary patterns of women in the study remain fairly constant (Northstone and Emmett, 2008), so it is likely that the women who did not consume fish in late pregnancy had continued this habit throughout the pregnancy.

### 4.3 Comparison with other studies

This is the second study that has assessed the associations of mercury with a cognitive outcome with stratification for maternal fish eating. Previous studies of fish, mercury and cognition have often analyzed the offspring outcomes by allowing for both mercury and fish as independent factors in the analyses (e.g. Lederman et al., 2008) but this is an over-control since the two variables are collinear − and it is well known that can result in some regression coefficients to have the wrong sign (www.stat.tamu.edu/∼hart/652/collinear.pd accessed 6th September 2016). For this reason the data have been stratified by fish consumption and evidence of interaction sought in both the INMA study (Llop et al., 2016) and our own. Their study was concerned with a high fish consuming population, only 6% of whom ate less than three fish meals a week. Even though this group was small, they were able to show that the relationship between the child’s cognitive function and cord blood mercury was different − in the 94% that were eating at least three fish meals a week the relationship between mercury and cognition was positive, but that it was negative in the 6% who ate less fish. The only other study to our knowledge that has assessed the relationships of mercury and outcome within non-fish eaters separately from fish eaters looked at blood pressure among women: it showed differing effects between the two, with increasing systolic blood pressure with increasing mercury in the non-fish eaters but the reverse among the fish eaters (Vupputuri et al., 2005).

The question often arises as to the sources of mercury among the non-fish eaters. It is a common fallacy that fish is the only source of mercury. In a previous study using this cohort we have shown that seafood contributes less than a half of the mercury obtained from the diet, which in turn contributes about 20% of blood mercury (Golding et al., 2013). Other sources include dental amalgams and inhalation from the atmosphere.

The major studies assessing prenatal mercury effects have often been based on populations with heavy seafood consumption and very few women eating no seafood (e.g. Faroes, Spain, Seychelles). Consequently a comparison of fish and non-fish eaters would not be possible. Of the three studies, one (in the Seychelles) has shown no relationship between mercury and cognition, one (in Spain) showed positive and the third (in the Faroes) found negative associations. One reason for the differences is that the populations of the Seychelles and Spain were eating fish, and those on the Faroes were eating sea mammals such as whale, which have a different nutrient pattern compared with fish. It is also notable that these studies estimated maternal mercury levels from the last trimester, whereas this study concentrates on the first half of pregnancy.

This is not the first study using ALSPAC to assess the link between prenatal mercury and IQ: an analysis of 1311 umbilical cords showed positive associations of mercury level with IQ that attenuated after adjusting for social and nutritional confounders (Julvez et al., 2013). Nevertheless, although not statistically significant the associations with mercury level remained positive for both verbal and full-scale IQ. This project used mostly different subjects from this study, with different measures with <20% overlap. Similar to this study, they found no adverse consequence on IQ when all subjects were analyzed together; differences between the children of mothers who did and did not eat fish were not assessed.

### 4.4 Possible explanations for our findings

If our findings are confirmed, they raise the question as to what the possible protective effect of fish might be. There is evidence that low fish intake is associated with low blood concentrations of vitamin D, choline and omega-3 fatty acids such as DHA (Wu et al., 2013), and that consumption of fish is associated with increases in blood selenium and urinary iodine (Bath et al., 2013). There has been at least one study demonstrating prenatal serum measures of vitamin D deficiency to be associated with poor performance on some neurodevelopmental measures (Whitehouse et al., 2012) and a suggestive result for prenatal choline intake in the Project Viva study (Boeke et al., 2013); in contrast one study using prenatal blood measures of omega-3 fatty acids in ALSPAC showed very little association with offspring IQ (Steer et al., 2013), and a review of randomized controlled trials of fish oil supplementation during pregnancy found no consistent effect on measures of cognition in the children (Campoy et al., 2012). Although selenium is thought to be important in protecting against any effect of mercury (Berry and Ralston, 2008), there was no evidence in this study that there was any marked contribution of selenium level to mask an effect of mercury on offspring IQ. Iodine is a possible contender: the UK population has low levels of body iodine (Pearce, 2015), and offspring IQ is positively associated with maternal prenatal levels (Bath et al., 2013). A parallel hypothesis is that the beneficial effects of maternal fish consumption on the brain of the developing fetus reflect the mixture of nutrients in fish rather than one specific nutrient.

### 4.5 Relevance to other populations

We have previously published a comprehensive comparison of prenatal mercury levels in ALSPAC with those found in other countries (Taylor et al., 2014: page 19 and Table 3). The mean/median levels are slightly higher than reported for similar developed countries. Thus the median value for mercury in the present study was more than twice that found in the USA in women of child-bearing age based on NHANES data. The 90th and 95th centiles, however, were higher in the US study than in the present study. This difference is unlikely to be due to a difference in consumption of seafood as consumption is less in the USA than in the UK, and mercury levels in UK seafood are generally higher than those in the USA. There are no other published data on prenatal mercury levels in the UK to our knowledge.

### 4.6 Need for replication

Clearly there is a need to replicate these results with inclusion of separate analysis of non-fish eaters and fish eaters. It is not possible, however, to do this where the whole population consume fish, nor is it feasible in studies where the likely exposure to mercury has been calculated based on the pregnant mothers’ fish intake (e.g. Vejrup et al., 2016), nor where the number of participants is small. It is noteworthy in this respect that most studies in the literature have considerably fewer than 500 mother–child pairs and various studies have reported a wide range of mean population blood mercury levels. The mean mercury levels in ALSPAC are typical of those in developed countries (Taylor et al., 2014). If confirmed in other studies it is likely that the beneficial effects of maternal fish consumption on the brain of the developing fetus reflect a mixture of nutrients in fish, including iodine, vitamin D, selenium, and long-chain fatty acids, and that these effects outweigh any adverse effects of mercury.

### 4.7 In conclusion

The data in the present study indicate that there are no adverse effects of prenatal mercury levels provided the mother eats fish, but that there may be an adverse effect if the mother eats no fish (P for interaction <0.05). This suggests that guidelines need to be simplified and the benefits of fish consumption emphasised more clearly.

## Funding statement

The UK Medical Research Council and the Wellcome Trust (Grant ref: 102215/2/13/2) and the University of Bristol currently provide core support for ALSPAC. The assays of the maternal blood samples were carried out at the Centers for Disease Control and Prevention (CDC) with funding from the National Oceanic & Atmospheric Administration (NOAA), and the statistical analyses were carried out in Bristol with funding from NOAA and support from the Intramural Research Program of the National Institute of Alcohol Abuse & Alcoholism (NIAAA) of the National Institutes of Health. CMT was supported by a Wellcome Trust Career Re-Entry Fellowship (Grant ref: 104077/Z/14/Z). The funders had no involvement in the study design nor in the collection, analysis and interpretation of the data and the researchers worked independently from the funders.
