# Supplementary material for: Maternal prenatal blood mercury is not adversely associated with offspring IQ at 8 years provided the mother eats fish: A British prebirth cohort study
Source: Int J Hyg Environ Health. 2017 Oct;220(7):1161–7. doi: 10.1016/j.ijheh.2017.07.004 (PMC5584731; doi:10.1016/j.ijheh.2017.07.004)
Supplement: Supplementary file 1 [file mmc1.docx]

|  | **N** | **Median** | **Mean (SD)** | **R^2^%** |
| --- | --- | --- | --- | --- |
| **Maternal age** |  |  |  |  |
| <20 | 239 | 1.34 | 1.50 (0.74) |  |
| 20-24 | 813 | 1.57 | 1.79 (1.00) |  |
| 25-29 | 1531 | 1.90 | 2.11 (1.11) |  |
| 30-34 | 1019 | 2.12 | 2.29 (1.07) |  |
| 35+ | 311 | 2.18 | 2.44 (1.21) | 4.87 |
|  |  |  |  |  |
| **Parity** |  |  |  |  |
| 0 | 1622 | 1.97 | 2.22 (1.24) |  |
| 1 | 1249 | 1.86 | 2.02 (0.95) |  |
| 2 | 540 | 1.86 | 2.02 (0.90) |  |
| 3+ | 239 | 1.68 | 1.86 (0.95) | 0.96 |
|  |  |  |  |  |
| **Maternal Education** |  |  |  |  |
| A (lowest) | 673 | 1.54 | 1.75 (0.95) |  |
| B | 335 | 1.73 | 1.89 (1.01) |  |
| C | 1155 | 1.88 | 2.03 (1.03) |  |
| D | 802 | 2.05 | 2.29 (1.16) |  |
| E (highest) | 547 | 2.40 | 2.60 (1.18) | 5.98 |
|  |  |  |  |  |
| **Smoked mid-pregnancy** |  |  |  |  |
| Yes | 752 | 1.61 | 1.83 (0.97) |  |
| No | 2968 | 1.96 | 2.16 (1.11) | 1.50 |
|  |  |  |  |  |
| **Alcohol consumption (units) mid pregnancy** |  |  |  |  |
| Not at all | 1789 | 1.76 | 1.96 (1.05) |  |
| <1/week | 1228 | 1.95 | 2.17 (1.07) |  |
| 1-6 / week | 550 | 2.09 | 2.28 (1.06) |  |
| 1+ / day | 65 | 1.94 | 2.39 (1.56) | 1.44 |
|  |  |  |  |  |
| **Housing tenure** |  |  |  |  |
| Owned/mortgaged | 2695 | 2.00 | 2.20 (1.10) |  |
| Council rented (public housing) | 570 | 1.52 | 1.71 (0.96) |  |
| Other | 444 | 1.79 | 2.02 (1.18) | 2.61 |

**Maternal prenatal blood mercury is not adversely associated with offspring IQ at 8 years provided the mother eats fish: a British prebirth cohort study.**

Jean Golding, Joseph R Hibbeln, Steven M Gregory, Yasmin Iles-Caven, Alan Emond, Caroline M Taylor.

**Supplementary Table**

Each continuous variable is statistically significant at P for trend <0.0001
